# Draft Genome Sequences of Three “*Candidatus* Symbiopectobacterium” Isolates Collected from Potato Tubers Grown in New Zealand

**DOI:** 10.1128/mra.01148-22

**Published:** 2023-02-28

**Authors:** Luciano Nunes Leite, Sandra B. Visnovsky, Peter J. Wright, Andrew R. Pitman

**Affiliations:** a The New Zealand Institute for Plant and Food Research Limited, Christchurch, New Zealand; b The New Zealand Institute for Plant and Food Research Limited, Pukekohe, New Zealand; c Better Border Biosecurity‡; Indiana University, Bloomington

## Abstract

The draft genome sequences of three “*Candidatus* Symbiopectobacterium” isolates that were collected from New Zealand-grown potato tubers represent the first report of this proposed taxon in the Southern Hemisphere. Their symbiosis with insects and nematodes and their presence on plants may lead to new strategies for pest control and crop management.

## ANNOUNCEMENT

Members of the symbiont taxon “*Candidatus* Symbiopectobacterium” occur among nematodes of the genus *Howardula* (Tylenchida, Allantonematidae) and several insect species in the orders Hemiptera and Hymenoptera (1). Bacteria belonging to “*Ca.* Symbiopectobacterium” are closely related to those in the genera *Pectobacterium* and *Dickeya* (*Enterobacterales*), which are well-characterized plant-pathogenic bacteria that are not commonly associated with insects. To date, “*Ca.* Symbiopectobacterium” genomes are marked by genome erosion characteristic of the early stages of symbiosis. The abundance of pseudogenes and reduced size, compared with related species (1.5 to 4.5 Mb in “*Ca.* Symbiopectobacterium” versus ≥5 Mb in *Pectobacterium* and *Dickeya*), may contribute to their symbiotic relationships with nematode and insect hosts (1).

Here, we report the draft genome sequences of three “*Ca.* Symbiopectobacterium” isolates that were collected from potato tubers during a screening of potatoes grown throughout New Zealand for plant-pathogenic *Pectobacterium* and *Dickeya* species (2). Bacterial cultures were isolated from the margins of infected tuber samples and spread as serial dilutions on crystal violet pectate medium containing novobiocin (1% [wt/vol]) at 27°C for 48 to 72 h. Colonies inhabiting cavities within 48 h were transferred to Kings B medium and incubated at 27°C for 24 h. In pathogenicity assays on potato plants, these isolates produced small or no lesions (2).

Pure cultures of the proposed “*Ca.* Symbiopectobacterium” isolates were subsequently incubated in lysogeny broth at 28°C for 16 h. Genomic DNA was isolated from cultures using a DNeasy blood and tissue kit (Qiagen); the resulting DNA was used for the construction of a DNBseq general DNA library and sequencing with the DNBseq platform to generate 150-bp paired-end reads for each isolate (BGI Genomics, Hong Kong). The quality of the sequence reads was checked using FastQC (Babraham Bioinformatics, UK), and low-quality reads (scores of <Q30) were trimmed using Fastq-Mcf (https://github.com/ExpressionAnalysis/ea-utils/blob/wiki/FastqMcf.md). *De novo* assembly was performed with the edited sequence reads using SPAdes v3.10.1 (3) to assemble the trimmed paired-end reads into contigs. Contigs shorter than 500 bp were eliminated. The draft sequence for each isolate was annotated using PGAP (http://www.ncbi.nlm.nih.gov/genome/annotation_prok) as part of the submission to NCBI. The identity of the sequenced organisms was established by comparison with genomes of 3 isolates of “*Ca.* Symbiopectobacterium,” 25 of *Pectobacterium*, 4 of *Dickeya*, 1 of Escherichia, and 1 of Pseudomonas, assessing 720 core genes analyzed with EDGAR (4) (data not shown).

The isolates reported here were identified as *Dickeya* spp., based on 16S rRNA gene sequencing, until the recent proposal of the taxon “*Ca.* Symbiopectobacterium.” The genome sequences of these isolates suggested their reclassification within this group of symbionts and extended the host range of this purported taxon. Furthermore, these isolates were the first of this taxon to be cultured *in vitro*, removing “*Candidatus*” status. Culturing of *Symbiopectobacterium* strains from potato provides new insight into their evolution and ecological role in agricultural systems.

### Data availability.

Raw data, draft genome sequences, and corresponding read data are available in GenBank under the accession numbers listed in Table 1. The versions described in this paper are the first versions.

**TABLE 1:** Statistics for the three draft “*Candidatus* Symbiopectobacterium” genome sequences

Isolate designation	Host	Location and year of isolation	GenBank assembly accession no.	GenBank genome accession no.	SRA accession no.	No. of coding sequences	No. of scaffolds	*N*_50_^*a*^ (bp)	Size of longest scaffold (bp)	Total size (Mb)	Total no. of paired-end reads
NZEC127	Solanum tuberosum	New Zealand, 2006	GCA_025962675.1	JAGFPG000000000	SRR21114645	4,742	116	779,297	2,199,636	5.29	4,153,539
NZEC135	Solanum tuberosum	New Zealand, 2006	GCA_025962695.1	JAGFPF000000000	SRR21114644	6,956	1,588	49,637	317,736	6.69	4,158,124
NZEC151	Solanum tuberosum	New Zealand, 2006	GCA_025962655.1	JAGFPE000000000	SRR21114643	4,555	36	591,814	1,766,505	5.16	4,146,890

a *N*_50_, shortest contig at 50% of the total genome length.
